# Hemorrhagic Myocardial Infarction and Intramyocardial Hemorrhage: From Microvascular Damage to Emerging Therapeutic Targets

**DOI:** 10.3390/medsci14020296

**Published:** 2026-06-07

**Authors:** Valentin Chioncel, Anamaria-Georgiana Avram, Raluca Ciomag

**Affiliations:** 1 Department of Cardiology, University of Medicine and Pharmacy Carol Davila, 050474 Bucharest, Romania; valentin.chioncel@umfcd.ro (V.C.);; 2 Emergency Clinical Hospital Bagdasar-Arseni, 041915 Bucharest, Romania

**Keywords:** hemorrhagic myocardial infarction, intramyocardial hemorrhage, no-reflow, microvascular obstruction, cardiac magnetic resonance, reperfusion injury

## Abstract

Intramyocardial hemorrhage (IMH) is a severe form of post-ischemic microvascular injury that may occur after myocardial infarction, particularly in the setting of extensive ischemia and reperfusion. IMH is closely related to microvascular obstruction (MVO), larger infarct size, impaired left-ventricular (LV) function, and adverse clinical outcomes. Cardiovascular magnetic resonance (CMR), especially susceptibility-sensitive techniques such as T2* or R2* mapping, enables in vivo detection of hemorrhagic microvascular injury and may refine post-MI risk stratification. Mechanistic and translational studies suggest that erythrocyte degradation and infarct-core iron deposition may contribute to persistent inflammation, maladaptive healing, adverse remodeling, and possibly arrhythmogenic substrate formation. However, most clinical evidence remains observational, and IMH-guided management has not yet been prospectively validated. This narrative review summarizes current evidence on the pathophysiology, imaging diagnosis, prognostic significance, and emerging therapeutic implications of IMH, while highlighting unresolved questions regarding standardized imaging, clinical implementation, and future phenotype-directed therapies.

## 1. Introduction

Despite major advances in acute coronary reperfusion, a substantial proportion of survivors of acute myocardial infarction (MI) develop progressive left-ventricular (LV) dysfunction, chronic heart failure, or sudden cardiac death in the months to years after the index event. Traditional explanations have focused on infarct size and neurohumoral activation; however, contemporary imaging has clarified that the microcirculation inside the infarct zone can decisively influence healing and remodeling.

Persistent (“late”) microvascular obstruction (MVO) is detected in a majority of large infarcts and is strongly associated with major adverse cardiac events (MACE). In this high-risk setting, intramyocardial hemorrhage (IMH) is frequently present and appears to identify a phenotype with the worst outcomes [1,2,3,4,5,6].

The importance of hemorrhage after reperfusion was recognized more than five decades ago. In a seminal canine model of prolonged coronary occlusion followed by reperfusion, myocardial hemorrhage was accompanied by marked hemoglobin accumulation within the injured zone and was associated with infarct extension in a subset of animals, suggesting that hemorrhage is not merely cosmetic but may exacerbate tissue injury and limit salvage [7]. Modern cardiac magnetic resonance (CMR) has revived and refined these observations by allowing in vivo detection of IMH, quantification of its extent, and correlation with clinical outcomes [8,9,10].

However, IMH remains underappreciated in routine practice for several reasons. First, its diagnosis typically requires CMR with susceptibility-sensitive sequences (T2* or R2* mapping), which are not universally performed after MI. Second, IMH is tightly coupled to other microvascular injury markers (especially MVO), making it challenging to disentangle causality from association. Third, there is no established, targeted therapy for IMH, and therefore clinicians may consider it a prognostic label rather than an actionable finding [11,12,13].

This review provides a clinically focused synthesis of the current evidence base, drawing on meta-analytic data and contemporary large cohorts to clarify how IMH fits within the spectrum of microvascular injury, how best to diagnose it, why it matters prognostically, and which therapeutic strategies are emerging. Particular attention is given to the concept that infarct-core iron may represent a mechanistic driver of chronic remodeling, providing a rational target for intervention. This is a narrative review; the literature was identified through focused searches of major bibliographic databases and reference lists, and the reporting structure was informed by PRISMA 2020 where applicable [1,2,6,14,15].

### Literature Search Strategy and Evidence Selection

This manuscript was designed as a narrative review rather than a formal systematic review. Literature was identified through focused searches of PubMed/MEDLINE, Scopus, Web of Science, and Google Scholar, complemented by manual screening of reference lists from relevant reviews and major clinical or CMR-based studies. The last literature update for this revised version was performed in May 2026. Priority was given to original clinical studies, meta-analyses, large CMR-based outcome cohorts, mechanistic translational work, preclinical studies addressing infarct-core iron, and recent expert reviews.

The main search strategy combined terms related to IMH and hemorrhagic MI with terms related to microvascular injury, imaging, prognosis, and therapy. Representative search strings included: “intramyocardial hemorrhage” OR “intramyocardial haemorrhage” OR “hemorrhagic myocardial infarction” AND “microvascular obstruction” OR “no-reflow” OR “cardiac magnetic resonance” OR “T2*” OR “R2*” OR “iron” OR “deferiprone” OR “left ventricular remodeling” OR “major adverse cardiac events”. Eligible publications included original clinical studies, systematic reviews/meta-analyses, CMR methodology papers, mechanistic experimental studies, and recent expert perspectives relevant to IMH pathophysiology, diagnosis, prognosis, or therapy. Publications were excluded when they did not address myocardial infarction-related IMH or MVO, lacked relevant imaging/pathologic data, were duplicate reports, or provided only indirectly related mechanistic information. English-language full-text publications were prioritized for the final qualitative synthesis.

Duplicate records and clearly overlapping cohorts were not counted repeatedly; when multiple publications appeared to report substantially overlapping populations, the most comprehensive, methodologically detailed, or most recent report was preferentially cited. Because this is a narrative synthesis, no formal risk-of-bias scoring or quantitative pooling was performed beyond summarizing published meta-analytic results.

Study selection followed a simplified pathway: records were identified through database and reference-list searches, screened at title/abstract level for relevance to IMH, MVO, CMR, prognosis, or therapy, assessed at full-text level when potentially eligible, and retained for qualitative synthesis when they contributed clinically or mechanistically important information. The reporting structure was informed by PRISMA 2020 where applicable, but PRISMA was not formally applied because the review was not designed as a systematic review. Table 1 summarizes representative studies that shaped the evidence synthesis.

## 2. Definitions and Conceptual Framework

The term “hemorrhagic myocardial infarction” is often used to describe an ischemic infarct complicated by IMH (i.e., blood products within the infarcted myocardium). In practice, IMH is the specific imaging/pathologic feature, whereas “hemorrhagic MI” is the clinical label for the overall phenotype [6].

Microvascular injury after MI is heterogeneous. MVO (often called “no-reflow” when observed invasively) reflects failure of microvascular perfusion despite restoration of epicardial patency. IMH is generally thought to require severe microvascular disruption with capillary rupture, allowing erythrocytes to extravasate. Thus, IMH typically occurs within regions of MVO, but MVO does not invariably progress to IMH [6,19,20,21,22,23].

Contemporary CMR outcome studies increasingly stratify patients into patterns such as: (1) no MVO/IMH, (2) mild or transient microvascular injury, (3) non-hemorrhagic MVO, and (4) hemorrhagic MVO (IMH). This patterning matters because it appears to separate groups with meaningfully different clinical trajectories-an observation that challenges the long-held assumption that MVO alone necessarily identifies the highest-risk microvascular phenotype [2,3,6].

IMH and MVO are often used interchangeably in discussions of microvascular injury, yet they may represent different biological states. For example, recent large-cohort data suggest that MVO without IMH can be associated with outcomes similar to those without microvascular injury, whereas IMH is strongly linked to subsequent MACE [2,3]. This implies that the presence of IMH can refine post-MI risk stratification beyond conventional infarct size and LVEF.

However, the distinction between non-hemorrhagic MVO and hemorrhagic MVO should be interpreted with caution. Selected contemporary cohorts suggest that MVO without IMH may carry lower risk than hemorrhagic MVO, but non-hemorrhagic MVO should not be regarded as universally benign. Its prognostic meaning may depend on infarct size, ischemic time, reperfusion quality, CMR timing, imaging protocol, endpoint definitions, and residual confounding.

### 2.1. MVO and No-Reflow as Context for IMH

MVO and the angiographic no-reflow phenomenon are often the first clues that microcirculation has failed despite successful epicardial PCI. Pathologically, MVO reflects a mosaic of endothelial injury, edema-related capillary compression, distal atherothrombotic embolization, platelet/leukocyte plugging, and microthrombi. The consequences are clinically important: reduced wash-in of oxygen and nutrients to border-zone myocytes, impaired delivery of circulating reparative cells, and delayed clearance of necrotic debris. From the perspective of IMH, MVO is not simply a co-traveler but rather a permissive state that creates the conditions for capillary rupture [19,21,22,23,24].

Chan and colleagues summarize CMR-based observations that >60% of acute MIs exhibit late (persistent) MVO and that hemorrhage is present in approximately three-quarters of infarcts with late MVO, conferring >50% increased risk for MACE compared with late MVO without hemorrhage [14]. These epidemiologic relationships suggest a hierarchical model in which MVO identifies a high-risk microvascular state and IMH identifies the subgroup with additional iron-driven injury potential [4,5,6,14].

Importantly, however, recent outcome studies complicate this hierarchy by showing that not all MVO carries the same prognostic weight. In the Lechner cohort, MVO without IMH had event rates similar to those without microvascular injury, whereas IMH was strongly prognostic [2]. One plausible biological interpretation is that MVO detected on early CMR can sometimes represent microvascular “stunning” or transient perfusion delay that recovers without structural capillary rupture, while IMH represents irreversible structural disruption with long-term consequences. This concept reinforces the need to assess IMH directly rather than inferring its presence from MVO [2,3,6,25].

This finding is hypothesis-generating rather than definitive. MVO remains an important marker of microvascular injury, and additional standardized studies are needed to determine when MVO without IMH represents transient microvascular dysfunction versus clinically meaningful structural injury.

From a clinical workflow standpoint, these insights support a two-step assessment: (1) determine whether MVO/no-reflow is present (angiographic indices, ST-segment resolution, and LGE-CMR), and (2) if present, determine whether the infarct core is hemorrhagic (T2* mapping) [13,26].

### 2.2. Histopathology of IMH

At histology, IMH is characterized by erythrocyte extravasation into the interstitium of the infarct core, often in close association with areas of capillary destruction and microvascular obstruction. Over time, hemoglobin degradation products are sequestered in macrophages, and iron can deposit as hemosiderin or other iron-containing aggregates. These deposits can persist for months, creating a localized, chronic inflammatory niche [8,9,10,27,28].

Translational work summarized by Chan and colleagues proposes that this iron-rich milieu promotes lipid oxidation and foam-cell formation within the infarct core, ultimately leading to fatty degeneration of infarcted myocardium [14]. This concept is particularly relevant because lipomatous metaplasia has been linked to arrhythmogenic substrate in chronic infarcts, and it provides a biological bridge between an acute microvascular event (IMH) and late clinical outcomes (heart failure and ventricular arrhythmias). This process shares prognostic implications with arrhythmogenic cardiomyopathy (AC), although the cellular origin differs (myocyte apoptosis and fibro-fatty replacement in AC versus iron-driven macrophage lipid accumulation in IMH) [14,29,30,31].

From a mechanistic standpoint, the persistence of iron helps explain why early microvascular injury can have long-lived consequences even after MVO resolves. It also provides a tangible therapeutic target that is unique to hemorrhagic infarction [30,31,32,33].

These mechanistic concepts should be distinguished from clinically validated conclusions. Iron persistence, macrophage recruitment, lipid oxidation, foam-cell formation, and fatty transformation are supported mainly by preclinical and translational observations, whereas the more firmly established clinical evidence is the association of IMH with larger infarct size, adverse LV remodeling, lower LVEF, and increased MACE. A direct causal chain from IMH to late clinical events has not yet been proven in adequately powered prospective clinical studies.

## 3. Pathophysiology

**Figure 1:**
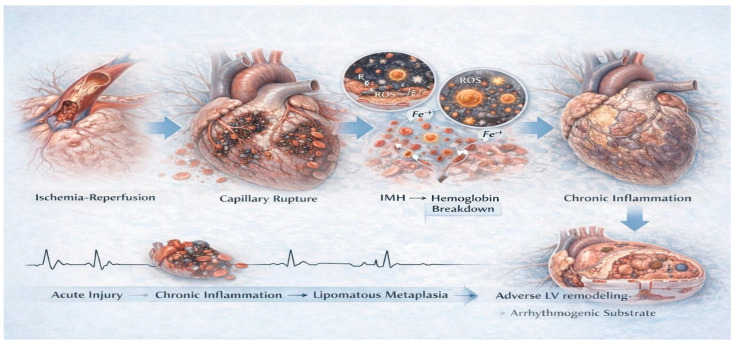
Pathophysiology of intramyocardial hemorrhage.

The microcirculation within the ischemic territory undergoes a time-dependent cascade of injury: endothelial swelling, glycocalyx disruption, leukocyte and platelet plugging, interstitial edema, and microthrombus formation (Figure 1) [21,22,24,34].

Reperfusion paradoxically exacerbates damage by subjecting compromised capillaries to restored hydrostatic pressure and shear stress, leading to capillary rupture and extravasation of erythrocytes into the infarct interstitium (IMH). Degraded hemoglobin releases redox-active iron (Fe^2+^/Fe^3+^), which catalyzes the Fenton reaction to generate reactive oxygen species (ROS). Persistent iron deposits recruit macrophages, sustain chronic inflammation, promote lipid peroxidation and foam-cell formation, induce ceroid accumulation, and drive macrophage apoptosis with iron recycling. These processes culminate in lipomatous metaplasia (fatty degeneration) of the infarcted myocardium, heterogeneous scar formation, adverse LV remodeling, impaired contractility, and increased susceptibility to ventricular arrhythmias and heart failure. Iron serves as the central modifiable substrate unique to hemorrhagic infarction, providing the mechanistic rationale for targeted chelation therapies [14,29,30,31,32,33,35].

Adverse remodeling after MI is multifactorial. Infarct size, duration of ischemia, reperfusion success, persistent MVO, endothelial dysfunction, neurohumoral activation, inflammatory signaling, mechanical wall stress, and scar maturation all contribute to the remodeling phenotype. Within this broader network, iron deposition should be interpreted as an additional mechanism that is distinctive to hemorrhagic infarction and potentially modifiable, not as the exclusive or dominant determinant of remodeling in every patient.

Classic experimental work provides an early mechanistic clue. Bresnahan and colleagues compared prolonged coronary occlusion with and without reperfusion in dogs and observed substantial hemoglobin accumulation within reperfused infarcts (reflecting hemorrhage). Importantly, a subset of reperfused animals showed infarct extension despite epicardial recanalization, implicating hemorrhage as a potential contributor to reperfusion-related injury rather than a benign secondary phenomenon [7,8,10,36].

After erythrocytes enter the infarct interstitium, hemoglobin is degraded to heme and iron-containing species. Iron can catalyze reactive oxygen species generation and lipid oxidation, and it may alter macrophage phenotype toward persistent inflammatory activation. CMR studies demonstrate that infarct-core iron can persist well beyond the acute phase, supporting the concept that IMH can seed a chronic injury signal rather than being a transient event [14,30,31,33].

**Figure 2:**
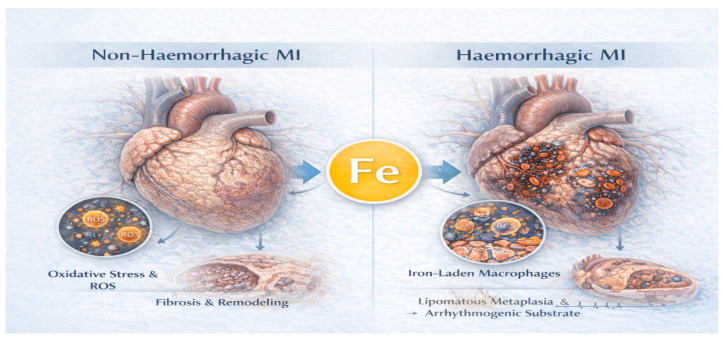
Central role of iron in hemorrhagic MI.

Recent mechanistic work has framed IMH as a potential contributor to chronic remodeling rather than as a solely descriptive imaging finding. In a microreview synthesizing translational data, Chan and colleagues argue that iron deposition within hemorrhagic infarcts may recruit macrophages to iron deposits and sustain a chronic inflammatory state. They propose an iron-driven cascade of lipid oxidation, foam-cell formation, ceroid production, foam-cell apoptosis, and iron recycling that could promote fatty transformation of infarcted tissue and impair LV function [14]—see Figure 2.

In this framework, IMH is best viewed as both a marker of severe microvascular injury and a plausible contributor to a distinct healing program, while recognizing that the degree of causality remains incompletely validated in clinical populations [14,29,32].

If infarct-core iron contributes to persistent inflammation and maladaptive remodeling, then chelating or otherwise neutralizing iron could represent a targeted investigational strategy. In the same translational narrative, deferiprone (an oral iron chelator) improved LVEF in preclinical hemorrhagic MI models compared with untreated hemorrhagic infarcts, and these observations have motivated a first-in-human trial (MIRON-DFP; NCT05604131) focused on hemorrhagic MI [14]. Definitive clinical efficacy remains unproven, and imaging improvement may not necessarily translate into better clinical outcomes without randomized validation [14,32].

Although IMH is most often discussed as an ischemia–reperfusion injury, it can occur in the absence of therapeutic reperfusion. In a prospective CMR study of patients with acute MI who did not receive reperfusion therapy, IMH was detected in 16 of 40 STEMI patients (and in only 3 of 41 NSTEMI patients). Importantly, the incidence of IMH among STEMI patients without reperfusion was comparable to contemporaneous STEMI patients undergoing primary PCI at the same center, suggesting that severe microvascular injury and hemorrhage may arise through prolonged ischemia and/or spontaneous reperfusion mechanisms [18]. This observation broadens the clinical context in which IMH might be relevant and cautions against equating IMH strictly with procedural reperfusion [8,9,18,27].

## 4. Diagnosis and Imaging

CMR uniquely provides a multi-parametric assessment of infarct anatomy and pathophysiology. Late gadolinium enhancement (LGE) defines infarct size and detects MVO as a hypoenhanced core within hyperenhanced infarct. T2-weighted approaches identify edema and, in principle, hemorrhage; however, susceptibility-based imaging (T2* or R2* mapping) is considered more specific for blood products and iron. Historically, MRI studies in the early 1990s and late 1990s already demonstrated post-reperfusion myocardial injury and hemorrhagic infarction in humans, anticipating the contemporary CMR era [6,13,26,37,38].

The central problem with conventional T2-weighted edema imaging is that signal changes reflect competing processes (edema, microvascular obstruction, and hemorrhage) that can cancel or mimic one another. A key methodological message reiterated in recent work is that T2* mapping is better suited to detect acute reperfusion hemorrhage than T2-weighted imaging, because paramagnetic blood products shorten T2* and increase R2* [3]. In practical terms, T2* mapping identifies IMH as a hypointense (or high R2*) core within the infarct [3,11,12,39,40,41,42].

IMH evolves dynamically. In a study of reperfused STEMI patients, IMH could be detectable as early as 1–2 days after reperfusion, and CMR performed around day 4–7 captures a high prevalence of IMH [16]. Other cohorts performed CMR at approximately 3 days after primary PCI, supporting that early subacute imaging is feasible for clinical phenotyping [17]. In routine practice, the optimal timing balances technical stability (patient condition, arrhythmia, renal function for gadolinium) with biological evolution (IMH and MVO maturation) [11,16,17,25].

A clinically useful approach is to classify post-MI microvascular injury into patterns based on LGE-defined MVO and T2*-defined IMH. In a contemporary cohort of 1109 STEMI patients undergoing CMR, Lechner and colleagues found that IMH (with MVO) identified a group with substantially higher risk of MACE, whereas MVO without IMH had outcomes similar to those without microvascular injury [2]. These results support adopting a phenotype-based report structure rather than reporting MVO alone—see Table 2.

Other modalities have roles, but are less specific. Invasive angiography can show no-reflow and microvascular dysfunction, but cannot directly identify hemorrhage. Echocardiography assesses LV function and mechanical complications, but cannot detect IMH. Computed tomography and nuclear methods have limited established roles for IMH. Biomarkers are non-specific: troponin reflects myocyte necrosis, and inflammatory markers lack spatial specificity. Therefore, when precise microvascular phenotyping is clinically important-e.g., for high-risk prognostication, trial enrollment, or mechanistic studies-CMR remains the reference.

Like emerging approaches, Ferumoxytol-enhanced T1-weighted CMR has shown promise in preclinical models for hyperacute detection of IMH (within hours of reperfusion) by exploiting the agent’s potent T1-shortening and extravasation into areas of microvascular disruption. Clinical translation is ongoing and may allow earlier phenotyping than standard T2* mapping [43].

### CMR Technical Considerations and Pitfalls

LGE is highly sensitive for infarct detection and can depict MVO as a dark core within hyperenhanced myocardium. However, LGE does not distinguish hemorrhagic from non-hemorrhagic MVO. For hemorrhage, susceptibility-sensitive techniques are needed.

T2* mapping measures the rate of signal decay related to magnetic field inhomogeneities; paramagnetic blood products shorten T2*, producing a low-signal core. Editorial perspectives emphasize that T2* mapping is generally superior to T2-weighted approaches for detecting acute myocardial hemorrhage, as T2 signal may be confounded by edema and by the presence of MVO itself [3].

Susceptibility effects increase with magnetic field strength; consequently, T2* values depend on scanner field (1.5T vs. 3T) and sequence parameters. Artifacts can arise from cardiac motion, arrhythmias, and local susceptibility interfaces (e.g., lung-heart boundary). In practice, robust breath-holding protocols, arrhythmia rejection, and careful post-processing are essential. Many studies define IMH based on T2* thresholds (commonly around <20 ms at 1.5T), but thresholds should be interpreted in the context of local protocol and normal reference values.

Edema peaks early and can evolve over the first week; MVO can be transient or persistent; IMH can emerge and expand in the first few days after reperfusion. These temporal dynamics mean that a single CMR snapshot may under- or over-estimate a given component depending on the imaging day. Clinical studies have therefore standardized imaging windows (e.g., day 3–7) to improve comparability [4,11,16,25].

A structured report for post-MI CMR should, at minimum, quantify infarct size, LV volumes and EF, and the presence/extent of MVO. When T2* mapping is available, the report should explicitly state whether IMH is present, its location (infarct core), and ideally its extent. Given emerging data that outcomes depend strongly on IMH status, the presence of IMH should be highlighted in the impression section rather than buried in technical details [2,13,26].

Protocol-dependent variability also limits generalizability. T2* and R2* values depend on field strength, pulse sequence, echo times, motion correction, and post-processing methods; therefore, thresholds such as T2* < 20 ms should be interpreted in the context of scanner field strength, local reference values, and acquisition timing. Serial T2*/R2* mapping may be useful for research and treatment-response assessment, but its routine clinical role remains unstandardized.

Despite its reference-standard role, widespread CMR implementation after STEMI is constrained by practical barriers. These include scanner availability, scheduling during the early post-MI window, patient instability, arrhythmias, limited breath-holding capacity, renal function considerations for gadolinium, variable availability of T2* protocols, local expertise in acquisition and interpretation, and uncertain cost-effectiveness of systematic screening.

## 5. Epidemiology and Determinants

Across reperfused STEMI cohorts, IMH is common. A systematic review and meta-analysis of 18 studies (n = 2824) reported an overall IMH prevalence of 39% after STEMI, while MVO was present in 61% [1]. Individual cohorts may report higher rates depending on infarct size, ischemic time, and CMR protocol. For example, in a reperfused STEMI cohort imaged at approximately 5.5 days, IMH was present in 54% of patients [16]. In a large modern cohort, IMH (with MVO) was found in 32% of STEMI patients [2].

Multiple predictors consistently point toward a model in which IMH marks a large ischemic burden and severe microvascular disruption. In the meta-analysis, IMH was associated with anterior infarction, larger infarct size, worse LVEF, and higher LV end-diastolic volumes; it was also linked to suboptimal epicardial reperfusion (post-PCI TIMI flow < 3) and to glycoprotein IIb/IIIa inhibitor use [1].

Detailed cohort analyses add granularity. In one reperfused STEMI cohort, the strongest predictors of IMH included anterior infarction and use of glycoprotein IIb/IIIa inhibitors, while hypertension showed an inverse association; extensive IMH was particularly associated with anterior infarction and glycoprotein IIb/IIIa inhibitor use [16].

The association between glycoprotein IIb/IIIa inhibitor use and IMH should be interpreted primarily as an observational association. These agents are often used in patients with high thrombus burden, impaired initial coronary flow, larger infarcts, and more severe STEMI presentations; therefore, confounding by indication is likely. Adjusted prospective studies or randomized data would be required to determine whether specific antithrombotic strategies independently modify IMH risk.

In another cohort imaged at ~3 days after primary PCI, pre-PCI TIMI 0 flow predicted IMH, and MVO size strongly correlated with IMH size (r ≈ 0.81), underscoring the pathophysiological coupling between the two phenomena [17].

A contemporary US real-world analysis of 24,181 revascularized STEMI patients identified hemorrhagic MI (hMI) in 23.8% using post-PCI troponin kinetics validated against CMR. Independent associations included male sex (adjusted RR 1.26), African American race (adjusted RR 1.15), active smoking, and untreated hypertension; treated hypertension was marginally protective [44]. These demographic and modifiable-risk-factor differences highlight opportunities for targeted prevention and underscore the need for inclusive trial enrollment.

Advanced CMR phenotyping is particularly valuable when troponin elevation may reflect type-2 myocardial injury rather than type-1 infarction (e.g., hypertensive emergencies), as it clarifies the specific substrate driving risk [45,46,47].

The dogma that IMH is a reperfusion injury is challenged by data in patients who did not undergo reperfusion therapy. In a prospective study of MI patients without reperfusion, IMH occurred frequently in STEMI (16/40) and infrequently in NSTEMI (3/41) [18]. While sample size limited statistical power for outcomes, the observation suggests that prolonged ischemia, spontaneous reperfusion, or microvascular collapse itself may be sufficient to produce hemorrhage.

A practical challenge in contemporary care is that myocardial injury can arise from diverse mechanisms beyond plaque rupture and reperfused STEMI. In hypertensive emergencies, for example, troponin elevation is common and may reflect myocardial injury or type 2 MI rather than type 1 MI. A systematic review of hypertensive emergencies found widely variable MI prevalence (3.6–59.6%) and myocardial injury prevalence (15–63%), highlighting the importance of standardized definitions and adequate biomarker assessment [46]. In a cohort of patients presenting with suspected MI, nearly half of those adjudicated as hypertensive crisis had myocardial injury, which was associated with higher 2-year mortality compared with non-injury [47]. While these studies do not address IMH directly, they emphasize why advanced phenotyping (including CMR when appropriate) matters: it can prevent misclassification and can clarify the specific substrate (necrosis, microvascular injury, hemorrhage) that drives risk [45,46,47].

## 6. Prognostic Significance

Across aggregated studies, IMH is consistently associated with worse outcomes. The meta-analysis by Vyas and colleagues found that IMH was linked to higher odds of MACE (pooled OR ≈ 2.63) [1]. IMH also tracked with structural and functional severity (larger infarct size, higher LV volumes, and lower LVEF), supporting its role as a marker of high-risk infarct biology [1,5,48,49].

A key recent advance is the separation of microvascular injury into patterns with distinct outcomes. In a cohort of 1109 STEMI patients, Lechner and colleagues compared outcomes across microvascular phenotypes and found that hemorrhagic MVO (IMH present) carried the highest risk: MACE occurred in 19.5% of patients with IMH versus 3.6% of those without microvascular injury. In multivariable analysis, IMH was independently associated with MACE (hazard ratio ≈ 3.88) [2]. Strikingly, non-hemorrhagic MVO did not show the same excess risk and had event rates similar to those without microvascular injury [2]. This finding suggests that not all MVO is biologically equivalent and that IMH may be the more potentially clinically relevant “red flag” within the microvascular injury spectrum.

The apparent incremental value of IMH over MVO alone should therefore be interpreted in the context of observational imaging data. IMH is closely linked to infarct size, ischemic time, pre- and post-PCI TIMI flow, MVO extent, LV volumes, and LVEF; residual confounding by the overall severity of ischemic and microvascular injury cannot be fully excluded.

These results align with the conceptual interpretation proposed in an accompanying editorial: MVO without IMH may represent potentially reversible microvascular stunning, whereas IMH reflects irreversible microvascular damage, with capillary rupture and an iron-laden infarct core that can perpetuate injury long after acute reperfusion [3].

Several cohorts suggest that IMH adds prognostic information after adjustment for conventional risk markers, but the degree of independence varies according to cohort selection, CMR timing, imaging definitions, endpoint composition, and statistical models. Future studies should test whether IMH improves risk prediction beyond infarct size, MVO extent, and LVEF using standardized acquisition protocols and externally validated prediction models.

In patients without therapeutic reperfusion, outcome data are more limited. In the prospective non-reperfused MI cohort, the MACE rate at 12 months was numerically higher in STEMI patients with IMH than in those without IMH (5/16 vs. 2/24, *p* = 0.063) [18]. Although not definitive, this trend supports the broader premise that IMH identifies a high-risk substrate regardless of whether reperfusion was procedural or spontaneous.

### 6.1. Clinical Implications

Taken together, the evidence supports three practical conclusions. First, when available, CMR detection of IMH can meaningfully refine risk stratification beyond infarct size and LVEF. Second, IMH should prompt heightened vigilance for adverse remodeling and heart-failure development, including early optimization of guideline-directed medical therapy and close follow-up. Third, IMH may identify the subgroup most likely to benefit from future targeted therapies (e.g., iron-modifying approaches) and therefore is an attractive enrollment criterion for mechanistic trials [6,30,31,32,50].

### 6.2. Key Studies in Context

Bresnahan and colleagues provided early evidence that hemorrhage may contribute to myocardial injury in the reperfusion setting. In a canine model of prolonged coronary occlusion (≈5 h) with or without reperfusion, they quantified intramyocardial hemoglobin and observed that reperfused infarcts had markedly higher hemoglobin content than non-reperfused infarcts, consistent with significant hemorrhage. Notably, while reperfusion salvaged myocardium in some animals, others exhibited infarct extension despite reperfusion, and hemorrhage was prominent in these lesions [7]. Although the experimental techniques of the era could not dissect microvascular mechanisms at the cellular level, the study foreshadowed two ideas that remain central today: (i) hemorrhage is a frequent consequence of reperfusion of severely ischemic tissue, and (ii) hemorrhage can co-occur with, and possibly amplify, irreversible injury.

The systematic review and meta-analysis by Vyas and colleagues aggregated 18 clinical studies (n = 2824) and reported IMH in 39% of reperfused STEMI patients, with MVO present in 61% [1]. Beyond prevalence, the analysis established a consistent association between IMH and worse LV structure/function and higher odds of MACE (pooled OR ≈ 2.63). The meta-analysis also identified clinical correlates such as anterior infarction, suboptimal angiographic reperfusion, and glycoprotein IIb/IIIa inhibitor use [1]. These pooled observations are valuable for external validity, but the analysis necessarily inherits heterogeneity in imaging protocols (T2 vs. T2* definitions), timing, and MACE definitions across the included studies [1,11,12,39].

Lechner and colleagues advanced the field by separating patients into microvascular injury patterns and directly comparing outcomes across these phenotypes. In 1109 STEMI patients undergoing CMR, they reported IMH (coexisting with MVO) in 32% and non-hemorrhagic MVO in 25% [2]. Crucially, hemorrhagic MVO carried markedly higher MACE rates (≈19.5%) compared with no microvascular injury (≈3.6%), and IMH remained independently prognostic (HR ≈ 3.88) [2]. Conversely, non-hemorrhagic MVO did not demonstrate comparable excess risk. This observation suggests that separating MVO into hemorrhagic and non-hemorrhagic forms may be clinically more informative than reporting MVO alone. The accompanying editorial interpretation that MVO without IMH may represent “microvascular stunning” further reinforces this biological stratification [3].

### 6.3. Predictors and Timing: Practical Lessons from Serial Imaging Cohorts

In a reperfused STEMI cohort imaged at ≈5.5 days, Amier and colleagues found IMH in 54% and demonstrated that it can be detected as early as 1–2 days after reperfusion [16]. They reported strong associations with anterior infarction and glycoprotein IIb/IIIa inhibitor use. Such findings are clinically useful because they identify patient profiles in which early CMR phenotyping is most likely to reveal IMH. Ma and colleagues, performing imaging evaluation of patients at ≈3 days post-PCI, showed that pre-PCI TIMI 0 flow predicted IMH and that IMH extent tracked closely with MVO size (r ≈ 0.81), emphasizing the tight biological linkage between capillary non-perfusion and capillary rupture [11,16,17].

Li and colleagues prospectively enrolled MI patients who did not receive reperfusion therapy and demonstrated that IMH can occur in this setting, particularly in STEMI (16/40 vs. 3/41 NSTEMI) [18]. The observation that IMH incidence in non-reperfused STEMI was comparable to that in contemporaneous reperfused STEMI at the same center challenges the simplistic label of IMH as purely an iatrogenic reperfusion complication. Instead, IMH may reflect the combined effects of prolonged ischemia, endogenous lysis/spontaneous recanalization, and microvascular structural failure.

The Cell Stress microreview synthesizes a mechanistic framework in which IMH introduces iron that can persist, recruit macrophages, sustain inflammation, and drive fatty degeneration of the infarct core, thereby contributing to chronic heart failure progression. The same translational narrative reports that deferiprone improved LV function in hemorrhagic MI models and that these findings have motivated a first-in-human trial [14]. While such translational data cannot substitute for randomized clinical outcomes, they provide a coherent mechanistic bridge between the acute imaging phenotype and late clinical risk [14,30,31,32].

### 6.4. IMH and Arrhythmogenic Substrate

Most clinical discussions of IMH focus on heart failure and remodeling, but several lines of reasoning suggest that IMH may also increase arrhythmic risk. First, IMH is associated with larger infarcts and worse LV function, both established predictors of ventricular arrhythmias. Second, persistent iron deposition may create localized electrical heterogeneity by sustaining inflammation and promoting non-uniform scar maturation. Third, the proposed pathway from hemorrhage to lipomatous transformation of infarcted tissue provides a plausible structural substrate for re-entry circuits [1,14,29,30,31,48,51].

This relationship should be regarded as biologically plausible and hypothesis-generating. Current clinical evidence most strongly supports associations between IMH and larger infarct size, adverse remodeling, lower LVEF, and MACE, whereas direct evidence linking IMH specifically to malignant ventricular arrhythmias remains limited.

These concepts may have practical implications in selected patients. Patients with hemorrhagic MI and low or borderline EF, palpitations, syncope, frequent ventricular ectopy, or marked remodeling may reasonably undergo closer rhythm surveillance during the remodeling window. However, ambulatory rhythm monitoring should be individualized and should not be presented as an IMH-specific guideline requirement; device therapy decisions remain anchored in established EF-based and clinical indications.

## 7. Clinical Management

At present, there is no guideline-mandated IMH-specific therapy. Therefore, management is best framed as (1) optimizing evidence-based post-MI care, (2) intensifying surveillance in high-risk phenotypes, and (3) considering referral for trials when available [6,50].

No prospective randomized study has yet demonstrated that IMH-guided management improves clinical outcomes. Accordingly, the following management considerations should be interpreted as a risk-aware clinical framework for selected high-risk patients rather than as a validated standard-of-care pathway.

Patients with IMH typically have large infarcts and high remodeling risk; therefore, aggressive implementation of guideline-directed medical therapy for secondary prevention and heart-failure prevention is essential. This includes high-intensity lipid lowering, antiplatelet therapy as indicated, beta-blockers, renin-angiotensin system inhibition (ACE inhibitor/ARB/ARNI), mineralocorticoid receptor antagonists and SGLT2 inhibitors when criteria are met, and careful blood-pressure and glycemic control. These therapies are not IMH-specific, but their absolute benefit is greatest in high-risk phenotypes [50].

In selected patients with large STEMI or high suspicion of severe microvascular injury, early subacute CMR (e.g., days 3–7) may be considered when clinically feasible and locally available. Potential triggers include prolonged ischemic time, pre-PCI TIMI 0 flow, large anterior infarction, incomplete ST-segment resolution, or early severe LV dysfunction. CMR reporting should explicitly state whether IMH is present (based on T2* or susceptibility mapping) and quantify MVO and infarct size. Repeat imaging (echocardiography at 6–12 weeks) can assess remodeling and guide ICD/CRT consideration according to standard criteria [13,16,17].

Because IMH may contribute to a chronic arrhythmogenic substrate through iron persistence and subsequent infarct remodeling, clinicians should maintain a low threshold for ambulatory rhythm monitoring in symptomatic patients or those with borderline EF. Early referral to cardiac rehabilitation and careful diuretic titration are often needed, as these patients may develop heart-failure symptoms during early remodeling [14,29,30,31].

Translational rationale supports the concept that iron may be a modifiable contributor in hemorrhagic MI. A first-in-human trial evaluating deferiprone in hemorrhagic MI (MIRON-DFP; NCT05604131) has been initiated [14]. Such strategies remain investigational; awareness of trial availability is important because IMH may serve as a phenotype-defining inclusion criterion for future mechanism-based studies [14,32,52].

### 7.1. Therapeutic Landscape and Emerging Targets

Multiple strategies have attempted to limit microvascular injury at the time of reperfusion, including mechanical or ischemic post-conditioning approaches and pharmacologic interventions targeting oxidative stress, inflammation, platelet activation, or microvascular tone. Despite biologic plausibility, a consistent reduction in hard clinical outcomes has been elusive. Chan and colleagues note that several mechanical/post-conditioning approaches to reduce late MVO have shown inconsistent benefit and that pharmacologic interventions at the time of reperfusion have not significantly reduced MACE in this high-risk subgroup [14]. These observations underscore that microvascular injury is multifactorial and that timing, patient selection, and mechanistic targeting matter [19,23,34].

The association between glycoprotein IIb/IIIa inhibitor use and IMH has been observed in both meta-analytic and cohort studies [1,16], but causality should not be inferred. Glycoprotein IIb/IIIa inhibitors are preferentially administered in highly thrombotic, high-risk STEMI presentations, which themselves are prone to distal embolization, impaired microvascular perfusion, and IMH. This confounding by indication should be acknowledged when interpreting observational associations. Future studies should evaluate antithrombotic strategies after rigorous adjustment for infarct severity, thrombus burden, ischemic time, baseline flow, and reperfusion quality.

Iron chelation is a mechanistically specific but experimental strategy for hemorrhagic MI because it targets a substrate introduced by hemorrhage itself. Translational data suggest that deferiprone improved LVEF in preclinical hemorrhagic MI models. The first-in-human MIRON-DFP trial (NCT05604131) is an ongoing randomized pilot evaluating deferiprone versus placebo in CMR-confirmed hemorrhagic MI; the primary endpoint is reduction in hemorrhagic-zone iron burden on serial CMR at 6 months. Even if imaging endpoints improve, clinical benefit for LV remodeling, heart-failure outcomes, arrhythmia reduction, or survival will require confirmation in adequately powered trials [14,29,32,52].

Safety considerations are central in post-MI patients. Deferiprone and other iron-targeting strategies require monitoring for neutropenia/agranulocytosis, hepatic dysfunction, renal considerations, gastrointestinal intolerance, infection risk, and potential drug interactions. Particular caution is needed in patients receiving antithrombotic therapy or those with renal or hepatic impairment. Until randomized safety and efficacy data are available, iron chelation should not be considered routine post-MI therapy outside clinical trials.

From a trial design perspective, the most plausible near-term endpoints include attenuation of LV remodeling (LV volumes, EF), reduction in infarct-core iron burden on serial T2* mapping, and improvement in clinical events in enriched high-risk IMH cohorts. Given that hemorrhagic MI represents a relatively large subgroup (~30–40% of reperfused STEMI) [1,2], adequately powered trials should be feasible if imaging-based screening is implemented.

### 7.2. Proposed Clinical Algorithm

The following algorithm is proposed as a conceptual clinical/research framework for selected high-risk STEMI patients, not as a guideline-mandated or prospectively validated standard of care. Decisions regarding early CMR, intensified follow-up, rhythm monitoring, and trial referral should be individualized according to patient stability, renal function, arrhythmia burden, local CMR availability, and existing post-MI guideline recommendations.

**Step 1**. Identify patients at high risk of microvascular injury: large anterior STEMI; prolonged ischemic time; pre-PCI TIMI 0 flow; incomplete ST-segment resolution; high thrombus burden (with or without glycoprotein IIb/IIIa inhibitor use); cardiogenic shock; and/or early echo showing severe dysfunction.

**Step 2**. Perform early subacute CMR when feasible (≈day 3–7) with LGE and T2* mapping. Classify the patient into a microvascular phenotype: (A) no MVO/IMH; (B) MVO without IMH; (C) IMH (hemorrhagic MVO).

**Step 3**. Escalate follow-up intensity for IMH phenotype: early post-discharge visit (1–2 weeks), rapid optimization of neurohumoral therapy, and planned imaging reassessment (echo at 6–12 weeks). Consider ambulatory rhythm monitoring in those with low or borderline EF or symptoms.

**Step 4**. Consider trial referral for iron-targeted or other phenotype-directed interventions when available.

**Figure 3:**
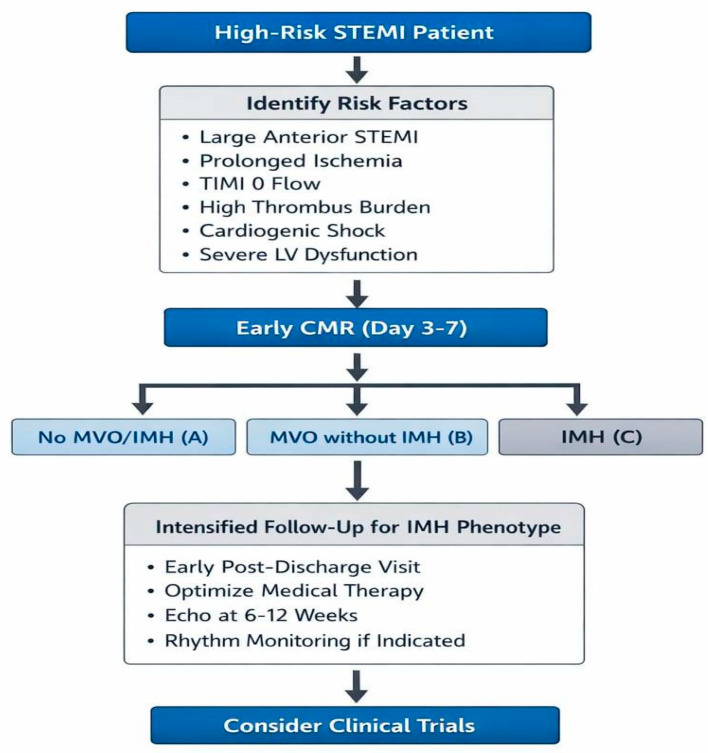
Proposed clinical/research framework for identifying IMH in high-risk STEMI patients.

This framework reflects current evidence suggesting that IMH may identify a subgroup with high downstream clinical risk, while acknowledging that IMH-guided management has not yet been prospectively validated and that non-hemorrhagic MVO remains clinically relevant—see Figure 3 [1,2,3,6,16,17].

Implementation of this phenotype-driven strategy may be limited by healthcare resource use, cost-effectiveness uncertainty, inter-center variability in T2*/R2* acquisition and interpretation, limited availability of CMR in the early post-MI window, and the absence of formal guideline recommendations supporting routine IMH-guided management.

## 8. Knowledge Gaps and Future Directions

Despite strong prognostic associations, several questions remain unresolved (Table 3).

**(1)** Causality vs. marker status: Even though mechanistic work supports a causal role for iron deposition in chronic remodeling, large clinical datasets still largely provide associative evidence. Serial, multi-timepoint CMR studies that track the evolution of MVO, IMH, and iron persistence in parallel with biomarkers and clinical outcomes will help resolve causal pathways [14,29,32].

**(2)** Standardization of CMR protocols and definitions: IMH detection depends on sequence selection (T2* mapping vs. T2-weighted imaging), field strength, and thresholds. Standardized acquisition and reporting recommendations-analogous to those developed for infarct size and MVO-would improve comparability across studies and facilitate multicenter trials [11,12,13,26,39].

**(3)** Integration into clinical decision pathways: It remains uncertain how best to integrate IMH into routine post-MI care pathways, including the cost-effectiveness of systematic CMR phenotyping and the role of IMH in deciding on intensity of follow-up, rehabilitation, and early heart-failure prevention strategies [6,50].

**(4)** Therapeutic targeting: Iron-targeted strategies are promising but unproven. Future trials will need to define the optimal therapeutic window, dosing, and safety monitoring for iron chelation in post-MI patients-many of whom receive antithrombotic therapy and may have borderline renal or hepatic function [32,52].

**(5)** IMH in non-reperfused and non-ST elevation syndromes: Evidence that IMH occurs without therapeutic reperfusion [18] raises new questions about its mechanisms and prevalence in delayed presenters, patients managed conservatively, and those with spontaneous recanalization. The biological meaning of small IMH foci in NSTEMI, and whether they carry the same prognostic weight as hemorrhagic reperfused STEMI, are not yet clear [18].

**(6)** Cost-effectiveness of early CMR phenotyping: Health-economic data specific to early CMR phenotyping after STEMI remain limited. However, a focused UK cost analysis in patients presenting with unexplained acute myocardial injury and culprit-free coronary angiography suggested that routine CMR increased costs in year 1, became cost-neutral by ~7 years, and was modestly cost-saving by 10 years, primarily by improving diagnostic accuracy and targeting downstream therapies. Whether similar economics apply to routine early CMR phenotyping of microvascular injury after large STEMI warrants formal evaluation alongside ongoing mechanistic trials [52,53].

## 9. Conclusions

IMH is common after MI and identifies a severe microvascular injury phenotype. Contemporary CMR studies suggest that IMH may provide incremental prognostic information beyond infarct size, LVEF, and MVO in selected cohorts, but current evidence remains largely observational and susceptible to residual confounding by infarct size, ischemic time, reperfusion success, baseline LV function, and overall severity of microvascular injury. Mechanistic and translational studies support a plausible role for infarct-core iron in persistent inflammation and maladaptive healing, but a direct clinical causal chain has not been definitively proven.

IMH-specific therapies are not currently established. Iron-targeted interventions, including deferiprone, remain investigational and require prospective validation for safety, imaging effects, LV remodeling, heart-failure outcomes, arrhythmias, and survival. In current practice, recognition of IMH should support risk awareness, optimization of guideline-directed post-MI therapy, individualized follow-up, and consideration of clinical-trial referral when available.

**Key Points**

• IMH represents extravasation of blood products into the infarcted myocardium and usually reflects severe microvascular injury. • CMR with susceptibility-sensitive sequences, particularly T2* or R2* mapping, is the most specific imaging approach for IMH detection. • IMH is associated with larger infarct size, worse LV function, adverse remodeling, and higher major adverse cardiac event (MACE) rates, although residual confounding remains possible. • No IMH-specific therapy is currently established; iron-targeted strategies remain investigational. • Future research should standardize imaging protocols, clarify incremental prognostic value, and test whether IMH-guided interventions improve clinical outcomes.


## Figures and Tables

**Table 1:** Selected evidence base for intramyocardial hemorrhage.

Study	Design/Population	IMH Assessment and Prevalence	Principal Relevance to this Review
Bresnahan et al., 1974 [7]	Canine coronary occlusion (~5 h) with or without reperfusion	Pathology; intramyocardial hemoglobin; prevalence not applicable	Early evidence that reperfused infarcts can become hemorrhagic and that hemorrhage may coexist with infarct extension.
Vyas et al., 2022 [1]	Systematic review/meta-analysis of reperfused STEMI (18 studies; n = 2824)	Varied CMR definitions using T2/T2*; IMH prevalence 39%	IMH associated with larger infarcts, lower LVEF, higher LV volumes, and higher odds of MACE.
Amier et al., 2017 [16]	Reperfused STEMI cohort	Subacute CMR (~5.5 days); T2*-based IMH; prevalence 54%	IMH could be detected early; anterior infarction and glycoprotein IIb/IIIa use were associated with IMH.
Ma et al., 2018 [17]	STEMI after primary PCI (n = 63)	CMR ~3 days; T2*-based IMH; prevalence 42.9%	Pre-PCI TIMI 0 predicted IMH; MVO size correlated strongly with IMH size.
Lechner et al., 2024 [2]	Large STEMI cohort with CMR phenotyping (n = 1109)	Subacute LGE-MVO and T2* IMH; IMH with MVO in 32%	IMH phenotype had substantially higher MACE and remained independently prognostic; non-hemorrhagic MVO findings require cautious interpretation.
Li et al., 2025 [18]	Prospective MI cohort without reperfusion therapy	CMR with T2*; IMH in 40% of STEMI (16/40)	Demonstrated that IMH can occur without therapeutic reperfusion, broadening the clinical context of hemorrhagic infarction.

**Table 2:** Diagnostic approaches for MVO and IMH after myocardial infarction.

Approach	Main Role	Strengths	Key Limitations/Technical Considerations
LGE-CMR	Defines infarct size and detects MVO as a hypoenhanced core within hyperenhanced infarct.	Widely used for infarct characterization and prognostic assessment.	Does not distinguish hemorrhagic from non-hemorrhagic MVO; requires gadolinium and may be limited by renal dysfunction or instability.
T2-weighted imaging/T2 mapping	Evaluates edema and area at risk; may show hypointense infarct core.	Useful for edema/infarct characterization and ischemic injury assessment.	Signal is confounded by edema, MVO, and blood products; reproducibility and thresholds vary by sequence, scanner, and timing.
T2* mapping	Susceptibility-sensitive detection of blood products and infarct-core iron.	Most specific routine CMR approach for IMH; common acute IMH thresholds are around T2* < 20 ms at 1.5T, depending on protocol.	Strongly field-strength and sequence dependent (1.5T vs. 3T); vulnerable to motion, arrhythmia, breath-holding limitations, and local susceptibility artifacts.
R2* mapping/susceptibility-weighted or dark-blood T2* methods	Alternative susceptibility-based techniques for hemorrhage/iron detection.	May improve conspicuity of hemorrhagic cores and reduce some blood-pool artifacts.	Requires expertise, local validation, and standardized post-processing; limited availability across centers.
Ferumoxytol-enhanced CMR and other emerging approaches	Potential hyperacute IMH detection by exploiting contrast-agent extravasation and T1 effects.	Promising in preclinical/early translational models for earlier phenotyping.	Clinical validation, safety, availability, and regulatory considerations remain unresolved.
Angiography, echocardiography, CT, nuclear imaging, biomarkers	Assess epicardial flow, LV function, anatomy, perfusion, or systemic injury.	Helpful for clinical management and differential diagnosis.	Cannot directly identify intramyocardial hemorrhage with sufficient specificity; biomarkers lack spatial localization.

**Table 3:** Major unresolved questions in IMH research.

Unresolved Question	Current Uncertainty	Priority for Future Work
Standardized imaging definitions	T2*, R2*, T2-weighted, and susceptibility-based criteria vary by scanner, sequence, field strength, and analysis method.	Develop consensus acquisition, thresholds, segmentation, and reporting standards for multicenter studies.
Optimal timing of CMR	IMH and MVO evolve dynamically during the first days after reperfusion, and a single scan may misclassify extent or phenotype.	Compare day 1–2, day 3–7, and serial imaging strategies in relation to remodeling and outcomes.
Serial T2*/R2* mapping	The clinical value of tracking infarct-core iron over time remains uncertain outside research protocols.	Define reproducibility, minimal clinically important change, and treatment-response endpoints.
Incremental prognostic value	IMH correlates with infarct size, ischemic time, reperfusion success, MVO extent, and LVEF.	Validate whether IMH improves risk prediction beyond conventional markers in standardized models.
Therapeutic targeting	Iron chelation and other phenotype-directed strategies remain investigational, and imaging improvement may not equal clinical benefit.	Conduct randomized trials with safety monitoring and clinically meaningful endpoints.
Arrhythmogenic risk	Mechanistic plausibility is strong, but direct clinical evidence linking IMH to malignant arrhythmias is limited.	Combine CMR phenotyping with rhythm monitoring, scar characterization, and electrophysiologic outcomes.
Implementation and cost-effectiveness	Routine early CMR phenotyping may be limited by scanner access, expertise, patient instability, and cost.	Perform pragmatic and health-economic studies focused on high-risk STEMI populations.

## Data Availability

No new data were created or analyzed in this study.
